# A Mass Spectrometric Analysis Method Based on PPCA and SVM for Early Detection of Ovarian Cancer

**DOI:** 10.1155/2016/6169249

**Published:** 2016-08-23

**Authors:** Jiang Wu, Yanju Ji, Ling Zhao, Mengying Ji, Zhuang Ye, Suyi Li

**Affiliations:** ^1^College of Electrical Engineering and Instrumentation, Jilin University, Changchun 130061, China; ^2^First Hospital, Jilin University, Changchun 130021, China

## Abstract

*Background*. Surfaced-enhanced laser desorption-ionization-time of flight mass spectrometry (SELDI-TOF-MS) technology plays an important role in the early diagnosis of ovarian cancer. However, the raw MS data is highly dimensional and redundant. Therefore, it is necessary to study rapid and accurate detection methods from the massive MS data.* Methods*. The clinical data set used in the experiments for early cancer detection consisted of 216 SELDI-TOF-MS samples. An MS analysis method based on probabilistic principal components analysis (PPCA) and support vector machine (SVM) was proposed and applied to the ovarian cancer early classification in the data set. Additionally, by the same data set, we also established a traditional PCA-SVM model. Finally we compared the two models in detection accuracy, specificity, and sensitivity.* Results*. Using independent training and testing experiments 10 times to evaluate the ovarian cancer detection models, the average prediction accuracy, sensitivity, and specificity of the PCA-SVM model were 83.34%, 82.70%, and 83.88%, respectively. In contrast, those of the PPCA-SVM model were 90.80%, 92.98%, and 88.97%, respectively.* Conclusions*. The PPCA-SVM model had better detection performance. And the model combined with the SELDI-TOF-MS technology had a prospect in early clinical detection and diagnosis of ovarian cancer.

## 1. Introduction

The mortality of ovarian cancer ranks first in female genital malignancies; owing to the fact of being uneasy to find, the 5-year survival rate is only about 30% [1]. Studies show that if ovarian cancer patients can get early diagnosis, the survival rate can be raised to about 90% [2]. Thus, early diagnosis and treatment are critical for improving the patients' cure rate and prolonging their survival.

Surfaced-enhanced laser desorption-ionization-time of flight mass spectrometry (SELDI-TOF-MS) is a new technology in proteomics research. For the accurately and quickly screening of large numbers of proteins within cells and tissues to identify specific tumor markers, it has a specific advantage in the early diagnosis of tumors [3–5].

However, the raw MS data is highly dimensional and redundant. Therefore, it is an important task to extract the features and establish a classification model in the massive MS data analysis. Currently MS data analysis methods mainly include pattern matching algorithm [6], genetic algorithm [7], chi-square test [8], extended Markov blanket [9], principal component analysis [10], artificial neural network [11], partial least squares analysis [12], robust SVM [13], and some combination methods [14, 15], such as wavelet and ANN, PCA, and SVM, in which the combination of PCA and SVM method obtains best results. But the principal component analysis (PCA) is based on the minimum variance principle of reconstruction, leading to a lack of probabilistic model structure and high order statistics. Probability PCA (PPCA) restricts the factor loading matrix with a noise variance estimation using the principle components ignored by the traditional PCA and then obtains the optimal probability model through the estimated parameters by the expectation-maximization algorithm. Consequently, PPCA can find the direction of the principal components from the high-dimensional data more effectively and can obtain the outstanding features extraction efficiently [16]. Simultaneously, the performance of SVM generally outperforms that of other classifiers applied in nonlinear classification, including iterative thresholding algorithm, self-organizing map, and *k*-nearest neighbor algorithm [17].

According to the above analysis, we focused on the design of an automatic model using PPCA and SVM technique for the ovarian cancer identification from MS data. In order to examine the performance of our proposed method, we established a PPCA-SVM model to classify ovarian cancer automatically and compared its average prediction accuracy, sensitivity, and specificity with those of a traditional PCA-SVM model using the same clinical data set.

## 2. Material and Methods

### 2.1. Data Set

The clinical data set used in this study was provided by the FDA-NCI center. By using the serum samples obtained by National Ovarian Cancer Early Detection Program (NOCEDP) and gynecologic oncology clinic at Northwestern University (Chicago, IL, USA), the FDA-NCI center formed the clinical data set via ProteinChip weak cation exchange interaction chips (WCX2, Ciphergen Biosystems, Inc., Fremont, CA, USA) and SELDI-TOF-MS technology [18]. The clinical data set consisted of 216 SELDI-TOF-MS samples, including 121 samples from ovarian cancer patients and 95 samples from healthy people.

**Figure 1:**
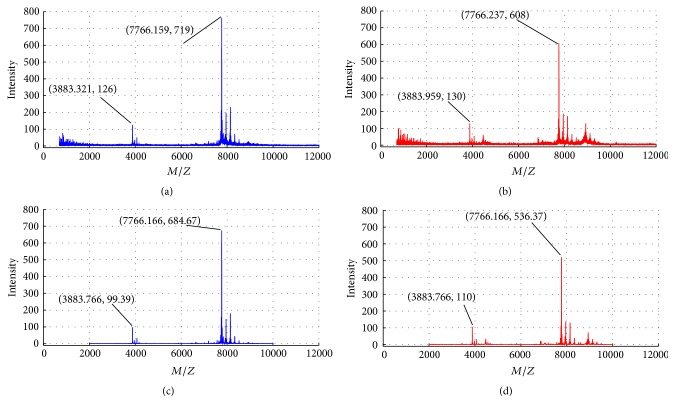
Comparison of SELDI-TOF-MS of serum from an unaffected individual (a) and from an ovarian cancer patient (b) and the corresponding preprocessing result of the unaffected individual (c) and that of the ovarian cancer patient (d).

The dimension of the raw SELDI-TOF-MS sample in feature space was high (each sample has about 360,000 features).  Figure 1(a) showed the spectrum of a healthy sample and Figure 1(b) showed that of an ovarian cancer patient. Differences could be seen in intensity of cancer sample and healthy sample. In Figure 1(a), the intensities were 126 and 719 at* M/Z* 3883.321 and 7766.159, respectively. In Figure 1(b), the intensities were 130 and 608 at* M/Z* 3883.959 and 7766.237, respectively.

From Figures 1(a) and 1(b), it can be seen that the valid information was concentrated between* M/Z* 2000 and* M/Z* 10000, and the raw spectrum contained a lot of redundancy and noise. Meanwhile, its prominent peaks needed to be aligned. Therefore, we employed the generally used preprocessing procedure to treat the raw data, including resampling, alignment, denoising, and normalization. The detailed description of the preprocessing procedure can be found in [5].  Figure 1(c) was the preprocessed spectrum of Figure 1(a) and Figure 1(d) was that of Figure 1(b). It can be seen that, after preprocessing, the dimension was reduced to 15000, the prominent peaks were aligned, the background was corrected, and the noise was suppressed.

### 2.2. Feature Extraction Using PPCA

After the preprocessing stage, the SELDI-TOF-MS data set was still highly dimensional. Extracting features by using dimension reduction techniques not only simplifies the structure of the prediction model but also improves the speed of training and testing. PCA is a commonly used dimension reduction technique based on the minimum variance principle of reconstruction. What is more, it uses the small amount of principle components to replace the massive data. However, PCA is lack of probabilistic model structure and highly order statistics. PPCA, proposed by Tipping and Bishop [16], restricts the factor loading matrix with a noise variance estimation using the principle components ignored by the traditional PCA in the latent variable model and then obtains the optimal probability model through the parameters estimated by the expectation-maximization (EM) algorithm. Consequently, PPCA can find the direction of the principal components from the high-dimensional data more effectively and can obtain the outstanding feature extraction more efficiently.

Suppose that the dimension of an observation data set {*S*_*n*_,  *n* = 1,2,…, *N*} is *d* and the number of samples is *N*. For one sample, through the latent variable model, the relationship between the observation data *S* and the latent variable *X* can be expressed as

(1) $$\begin{array}{c} S=WX+\mu +\varepsilon , \end{array}$$

where *W* is a *d* × *q* factor loading matrix, *X* is a *q*-dimensional latent variable, *μ* = (1/*N*)∑_*n*=1_^*N*^ *S*_*n*_, is a nonzero mean, *ε* is error and assume *X* ~ *N*(0, *I*) and *ε* ~ *N*(0, *σ* ^2^ *I*), and then we can obtain the probability distribution of *S* under the condition of *X* through (1) as follows:

(2) $$\begin{array}{c} p\left(\;\frac{S}{X}\;\right)={\left(\;2\pi \sigma^{2}\;\right)}^{-d/2}\exp\left\{\;-\frac{1}{2\sigma^{2}}{\left\|\;S-WX-\mu \;\right\|}^{2}\;\right\}. \end{array}$$

If the prior probability model of *X* conforms to Gaussian distribution

(3) $$\begin{array}{c} p\left(\;X\;\right)={\left(\;2\pi \;\right)}^{q/2}\exp\left\{\;\frac{1}{2}X^{T}X\;\right\} \end{array}$$

then the probability distribution of *S* can be expressed as

(4) pS=2π−d/2C−1/2exp⁡−12S−μTC−1S−μ,

where *C* = *WW* ^*T*^ + *σ* ^2^ *I* is a *d* × *d* matrix. By using Bayes rule, we can derive the posterior probability distribution of *X* from *S*:

(5) pXS=2π−q/2σ2M−1/2exp⁡−12S−μTM−1S−μ,

where *M* = *W* ^*T*^ *W* + *σ* ^2^ *I* is a *q* × *q* matrix. Under this model, the Log-likelihood function of *S* can be expressed as

(6) $$\begin{array}{c} L=-\frac{N}{2}\left\{\;d\ln\left(\;2\pi \;\right)+\ln\left|\;C\;\right|+tr\left(\;C^{-1}U\;\right)\;\right\}, \end{array}$$

where *U* = (1/*N*)∑_*n*=1_^*N*^(*S*_*n*_ − *μ*)(*S*_*n*_ − *μ*)^*T*^ is the covariance matrix of the observations, and then we can obtain the maximum likelihood estimates through the EM algorithm:

(7) W~=SWσ2I+M−1WTSW−1,

(8) σ~2=1dtrS−SWM−1W~T,

where *W* is the old value of the parameter matrix and $\tilde{W}$ is the revised estimates calculated from (7). We bring the parameters obtained from (7) and (8) into (1) to derive the latent variable ${\tilde{X}}_{n}$ which is the dimensionality reduction form of the observations *S*_*n*_:

(9) $$\begin{array}{c} {\tilde{X}}_{n}={\tilde{W}}^{T}\left(\;S_{n}-\mu \;\right). \end{array}$$

From (9), we can reconstruct the observation data ${\tilde{S}}_{n}$ via ${\tilde{X}}_{n}$:

(10) $$\begin{array}{c} {\tilde{S}}_{n}=\tilde{W}{\left(\;\tilde{W}W\;\right)}^{-1}{\tilde{X}}_{n}+\mu . \end{array}$$

### 2.3. SVM Model

SVM is derived from statistical learning theory. Its learning goal transforms empirical risk minimization into structure risk minimization and improves the overfitting problem [19]. In this study, the data set was under the PPCA dimensionality reduction procedure. And then we employed SVM technology to build an automatic detection model for ovarian cancer classification.

The implementation of the model establishment can be converted into solving the optimization as follows:

(11) Minimize 12wtw+c∑i=1NξiSubject  to ynwtϕxn+w0≥1−ξn,n=1,2,…,N,

where *x*_*n*_ is the dimensionality reduction data set after PPCA, *n* is the number of samples, *c* is a regularization constant, which determines the weigh between the maximum margin and the minimum classification error, *ξ*_*n*_ is the slack variable, *y*_*n*_ is the desired output, and *ϕ*(*x*_*n*_) is the kernel function that maps nonlinear data into linear in high-dimensional space.

### 2.4. Implementation of the PPCA-SVM Classifier

In this study we used MATLAB R2013 software and Lib-SVM toolbox [20] to build the classifier, and the implementation steps are as follows.

**Step 1 (selection of the training set and the prediction set).**

The preprocessed clinical data set included 216 samples; each sample had 15000 protein absorption features and had an appropriate type of clinical categories, negative for normal and positive for ovarian cancer patients.

We chose 70% of the data set randomly as the training set, the remaining as the prediction set.

**Step 2 (feature extraction).**

We used PCA to reduce the dimension. The cumulative contribution rate could reach 99.99% when using 215 principal vectors in PCA. So we applied PCA for feature extraction, reducing the data dimension from 15000 to 215 and PPCA for that using the same principal vectors.

**Step 3 (SVM modeling).**

We employed SVM to establish the detection model and trained the SVM model using a radial basis function (RBF) kernel, which maps nonlinear data into a higher dimensional space. In order to obtain the optimal combination of penalty parameters, *c* and *g* of the RBF kernel, we conducted 10-fold cross-validation based on the training set and then established SVM model by applying training set as input matrix and clinical categories as output matrix.

**Step 4 (model evaluation).**

The detection model was established by using the training set. We used the prediction set to verify its performance. The evaluation parameters included the prediction accuracy (Accuracy = ((TP + TN)/(TP + TN + FP + FN)) × 100%), the sensitivity (Sensitivity = (TP/(FN + TP)) × 100%), and the specificity (Specifity = (TN/(FP + TN)) × 100%), where TP, TN, FP, and FN were the number of true positive, true negative, false positive, and false negative, respectively. To avoid accidental error, this experiment was repeated for 10 times.

## 3. Results and Discussion

Using the prediction set, we conducted the prediction experiments for 10 times and compared the evaluation parameters of the PPCA-SVM model and the PCA-SVM model, respectively.  Table 1 showed the accuracy, sensitivity and specificity in classification.

Table 1 showed that the average prediction accuracy, the sensitivity, and the specificity of the PCA-SVM model were 83.34%, 82.70%, and 83.88%, respectively. In contrast, those of the PPCA-SVM model were 90.80%, 92.98%, and 88.97%, respectively. The PPCA-SVM model obtained higher accuracy, sensitivity, and specificity, outperforming the PCA-SVM model.

**Figure 2:**
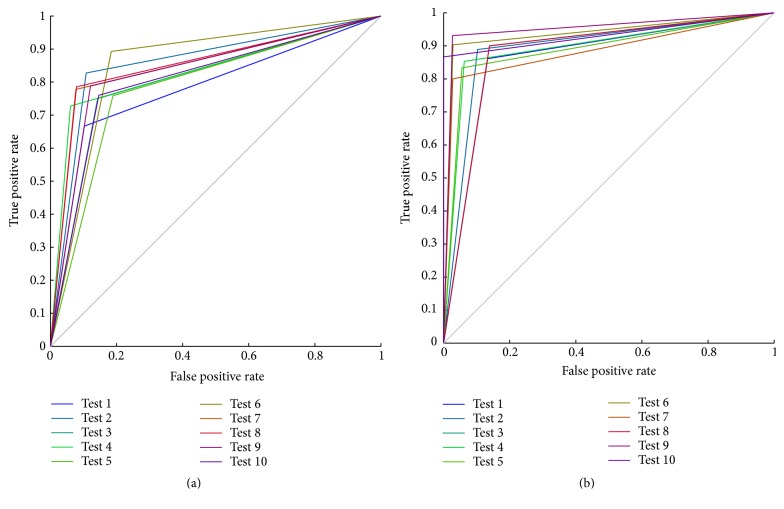
ROC graphic of PCA-SVM method (a) and ROC graphic of PPCA-SVM method (b).

To evaluate the accuracy of the classifier with binary outcomes, we also drew the receiver operating characteristic (ROC) curve of the PCA-SVM and the PPCA-SVM model, respectively.  Figure 2(a) showed the ROC curves obtained under 10 prediction experiments using the PCA-SVM classifier, and Figure 2(b) showed that using the PPCA-SVM classifier.

It is known that, in ROC space, the closer to the upper left corner, the higher the forecast accuracy. Oppositely, the closer to the bottom right corner, the lower the accuracy. Comparing the ROC curves of the PCA-SVM (Figure 2(a)) with that of the PPCA-SVM classifier (Figure 2(b)), the distance between the upper left corner and the ROC curves in Figure 2(a) was less than that in Figure 2(b), which meant the PPCA-SVM classifier was superior to the PCA-SVM classifier.

## 4. Conclusions

Early diagnosis of ovarian cancer can significantly improve the patients' cure rate and prolong their survival time. SELDI-TOF-MS has been shown to be an efficient technique in the early diagnosis of tumors, which enjoys large numbers of proteins screening within cells and tissues to identify specific tumor markers accurately. In this study, we used 216 SELDI-TOF-MS samples of ovarian cancer patients and healthy people to research an automatic detection method which enjoyed higher prediction accuracy and efficiency and propose a PPCA-SVM classifier. To verify the model, we compared the accuracy, sensitivity, specificity, and ROC of the PPCA-SVM and those of the traditional PCA-SVM classifier through numerous experiments. The results indicated that the PPCA-SVM model was an accurate and effective model to identify the ovarian cancer, and the PPCA-SVM method combined with the SELDI-TOF-MS technology had a prospect in early clinical diagnosis of cancer.

## Figures and Tables

**Table 1:** Comparison of the accuracy, sensitivity, and specificity of the PCA-SVM and of the PPCA-SVM model.

	PCA-SVM prediction (%)			PPCA-SVM prediction (%)		
	Accuracy	Sensitivity	Specificity	Accuracy	Sensitivity	Specificity
1	80.30	81.81	79.54	87.87	87.50	88.09
2	86.36	85.71	86.84	89.39	88.88	89.74
3	81.81	75.00	85.71	86.36	93.33	80.55
4	83.33	92.30	77.50	90.90	90.62	91.17
5	78.78	75.86	81.08	89.39	92.59	87.18
6	84.84	78.12	91.17	93.93	96.55	91.89
7	86.36	87.50	85.71	89.39	96.00	85.36
8	86.36	88.00	85.36	92.42	88.88	94.87
9	83.33	86.66	80.55	92.42	95.45	90.90
10	81.81	76.00	85.36	93.93	100	90.00

Average	83.34	82.70	83.88	90.80	92.98	88.97

## Abbreviations

SELDI-TOF-MS: Surfaced-enhanced laser desorption-ionization-time of flight mass spectrometry
PCA: Principal components analysis
PPCA: Probabilistic principal components analysis
SVM: Support vector machine
NOCEDP: National ovarian cancer early detection program
EM: Expectation-maximization
RBF: Radial basis function
TP: True positive
TN: True negative
FP: False positive
FN: False negative
ROC: Receiver operating characteristic.
